# Pioneering the IBD specialist nurse role in China: a shared decision-making model for clinical practice innovation

**DOI:** 10.3389/fmed.2025.1635290

**Published:** 2025-08-25

**Authors:** Shuyan Li, Zijun Ni, Yan Ma, Yan Chen, Hongling Sun, Jingfen Jin

**Affiliations:** ^1^Department of Nursing, The Second Affiliated Hospital, Zhejiang University School of Medicine, Hangzhou, China; ^2^Center for Inflammatory Bowel Diseases, Department of Gastroenterology, The Second Affiliated Hospital, Zhejiang University School of Medicine, Hangzhou, China; ^3^Key Laboratory of the Diagnosis and Treatment of Severe Trauma and Burn of Zhejiang Province, Hangzhou, China; ^4^Zhejiang Provincial Clinical Research Center for Emergency and Critical Disease, Hangzhou, China

**Keywords:** shared decision-making, nurse, inflammatory bowel disease, IBD, clinical practice

## Abstract

**Background:**

Inflammatory bowel disease (IBD) is a chronic condition characterized by the need for highly individualized treatment plans, requiring patients to make numerous complex medical decisions. Shared decision-making (SDM) has proven effective in improving treatment outcomes, patient satisfaction, and adherence in IBD management; however, its clinical implementation remains challenging. In China, formal SDM nurse roles have not yet been established.

**Aim:**

To establish an IBD specialist nurse position to facilitate the effective implementation of SDM in IBD care, enhance patient engagement in disease management, and improve the overall quality of IBD care in China.

**Methods:**

Since March 2021, the Second Affiliated Hospital, School of Medicine, Zhejiang University has actively promoted the establishment of the IBD specialist nurse role. This initiative defined the organizational structure, selection criteria and processes, role functions, core competencies, and training and assessment standards for the position. Guided by the Ottawa decision support framework, the responsibilities of the IBD specialist nurse within the SDM model were clarified, offering new strategies and practical approaches for IBD patient management.

**Results:**

Between March 2021 and March 2024, the IBD specialist nurse managed 2,028 patient visits, including 1,352 cases of Crohn's disease and 676 cases of ulcerative colitis. These included patients who were newly diagnosed, perioperative, in the preconception stage or pregnant, receiving biological therapies, or requiring vaccinations. Decision conflict assessments in 121 perioperative patients demonstrated lower conflict levels compared to previous studies. “Annual team recognition” of the IBD nursing role reached 94.12%. Additionally, the IBD specialist nurse played a pivotal role in nursing discipline development by establishing clinical protocols, revising practice guidelines, enhancing patient education materials, and participating actively in quality improvement initiatives and research projects.

**Conclusion:**

Our center successfully established the IBD specialist nurse role within the SDM framework, resulting in reduced patient decision conflict, improved treatment adherence, and enhanced quality of life. Future efforts will focus on refining the role evaluation system, optimizing training and promotion strategies, and strengthening policy support to further advance the professional development and broader implementation of IBD specialist nurses, thereby improving IBD management and healthcare delivery.

## Highlights

- To meet the need for effective SDM among IBD patients, the role of the IBD specialist nurse was formally introduced in China in March 2021.
- This study outlined the responsibilities of IBD specialist nurses within the SDM framework and reports on their positive impact from 2021 to 2024.
- While IBD specialist nurses play a key role in SDM, challenges remain regarding evaluation systems and professional training; recommendations are provided for future development.

## 1 Introduction

Inflammatory bowel disease (IBD) is a chronic, lifelong condition characterized by the necessity for highly individualized treatment and disease management strategies. Consequently, patients with IBD are required to make numerous medical decisions, including in relation to treatment and nursing care, throughout the course of their illness. The shared decision-making (SDM) model refers to a collaborative process in which healthcare professionals engage in discussions with patients, taking into account their preferences, beliefs, and values. Through the exchange of information, patients are provided with a comprehensive understanding of the risks and benefits associated with various medical options, thereby facilitating informed decision-making (1). The core principle of SDM is patient autonomy, and the importance of patient involvement in the medical decision-making process is highlighted (2). SDM is regarded as the optimal model for delivering patient-centered care (2). Studies have demonstrated that SDM enhances treatment effectiveness, patient satisfaction, and adherence among individuals with IBD (3).

However, the practical implementation of SDM in clinical settings is often impeded by barriers such as limited consultation time and inadequate professional knowledge among healthcare providers, making it challenging to execute SDM effectively (4). In international practice, SDM nurses play a pivotal role within the SDM model by assisting patients in weighing the advantages and disadvantages of different options, facilitating satisfactory decision-making, and reducing decision conflict and regret (5). In contrast, China has yet to establish a formal role for SDM nurses. Some researchers have proposed that specialist nurses or advanced practice nurses could assume this responsibility (6).

Under the SDM model, the practice of IBD specialist nurses was primarily guided by the Ottawa decision support framework (ODSF) (7). Developed by O'Connor et al. the ODSF is based on sociological, psychological, and economic theories and aims to assist patients in making informed decisions through evidence-based methods. The framework's core components include decision needs, decision support, and decision outcomes (7).

In an effort to promote the effective implementation of SDM in the management of IBD, encourage active patient participation in disease management, and enhance the quality of IBD care, a position for an SDM specialist nurse was established at our institution in March 2021. This position, staffed by one full-time IBD specialist nurse, is designed to assist patients in clarifying their preferences, developing appropriate risk perceptions, reducing decision conflict, making value-congruent decisions, minimizing decision regret, and ultimately improving health outcomes. This study describes the establishment and practice of the IBD specialist nurse role within the SDM framework.

## 2 Methods

### 2.1 Establishment of the IBD specialist nurse position

#### 2.1.1 Organizational structure

In 2016, the Second Affiliated Hospital, School of Medicine, Zhejiang University, established a multidisciplinary IBD Diagnosis and Treatment Center, which became one of the first regional IBD treatment centers in China in 2019. The center comprised a multidisciplinary team, including IBD Internal Medicine, Surgery, Pathology, Radiology, Nutrition, Laboratory Medicine, Pharmacy, and Dermatology. In response to the demands of multidisciplinary care and comprehensive disease management for IBD, the IBD specialist nurse position was established in March 2021. This position was part of the hospital's full-time specialist nursing team and was jointly managed by the Department of Gastroenterology and the Nursing Department. Attendance and salary distribution were collaboratively overseen by the Department of Gastroenterology and the Nursing Department, while continuing education, specialty practice development, and professional title advancement were uniformly managed by the Nursing Department.

#### 2.1.2 Selection criteria and procedure

The selection of one full-time IBD specialist nurse was conducted through an internal recruitment process.

##### 2.1.2.1 Selection criteria

Candidates had to be N3-level (or higher) nurses (N0–N4, ascending rank, with higher numbers indicating higher ranks) with a bachelor's degree, ≥5 years of clinical experience (≥3 in IBD), or master's-prepared nurses with ≥3 years in IBD; hold the title of charge nurse or above (specialist certification preferred); possess ≥CET-4 English proficiency; and have participated in ≥1 nursing research project or published ≥1 first-author paper in the past 3 years.

##### 2.1.2.2 Selection procedure

The selection process followed a three-tier system: “Initial Screening by Department–Preliminary Review by Major Department–Final Evaluation by Nursing Department.” Upon approval by the Nursing Department, the full-time nurse manager organized training and assessment. Candidates who successfully passed the assessment were formally appointed to the position.

#### 2.1.3 Role functions and core competencies

In developing the IBD specialist nurse role, the institution aligned its framework with the guidelines of the Nursing Committee of the European Crohn's and Colitis Organization (N-ECCO) to ensure standardized practice implementation. These guidelines define key responsibilities, such as nursing assessments for IBD patients, medication management, perioperative and transitional care, reproductive health management, and the provision of psychological support (8).

Additionally, by referencing the roles, core competencies, and practices of SDM nurses internationally (9, 10), a preliminary definition of the role functions and core competencies for the IBD specialist nurse within the SDM model was established. The IBD specialist nurse served as a collector, healthcare provider, and sharer of information during the SDM process between healthcare providers and patients. The nurse also acted as a patient advocate and a provider of psychological support. This role required strong professional competencies and the ability to communicate risks effectively. Beyond mastering a comprehensive body of knowledge regarding IBD treatment options, the nurse was required to assess patients' value preferences during communication and engage in discussions about the uncertainties associated with decision-making.

Given the specialized nature of IBD care, and referencing the *Consensus on the Diagnosis and Treatment of Inflammatory Bowel Disease* (2018, Beijing) (11) and international research on chronic disease management for IBD (8), our hospital developed comprehensive management protocol for the full-course management of IBD patients. These management protocol clearly defined the job responsibilities of the IBD specialist nurse.

#### 2.1.4 Training and assessment

##### 2.1.4.1 Training of the IBD specialist nurse

Upon assuming the position, the IBD specialist nurse underwent comprehensive training based on international and national IBD diagnosis and treatment guidelines, as well as pharmaceutical advancements. The nurse participated in specialized IBD courses and received training in procedures such as biologic infusion, stoma care, and enteral nutrition tube insertion. Active involvement in multidisciplinary case discussions, particularly focusing on comprehensive management strategies for refractory IBD, was also required. In addition, the nurse was trained in new techniques, including capsule endoscopy and the key points of intestinal ultrasound assessment.

Training also covered the use of screening tools for common psychological issues in IBD patients, including anxiety, depression, and disease-related stigma. Moreover, the IBD specialist nurse completed more than 25 continuing education credits annually, including participation in national or provincial education courses, nursing rounds, professional learning sessions, and academic paper publications.

Our IBD specialist nurse completed specialized training for wound, stoma, and incontinence nursing in Zhejiang Province, earning a specialist nurse Certificate. Furthermore, the nurse completed the N-ECCO IBD Nurse School course in Germany and obtained a certificate of completion.

Additionally, the nurse underwent online SDM training through the Ottawa Hospital website (https://decisionaid.ohri.ca/index.html), focusing on key areas such as needs assessment, values clarification, and the application of decision aids. This training ensured that the IBD specialist nurse possesses the professional competencies necessary to engage in SDM with patients.

##### 2.1.4.2 Assessment of the IBD specialist nurse

Each quarter, the IBD specialist nurse submitted a detailed report to the Nurse Manager, Nursing Department, and Teaching Department. This report outlined activities related to IBD specialized nursing, patient education, clinical nurse training, specialty nursing research, and plans for the forthcoming quarter. At the end of each year, the nurse underwent an annual evaluation assessing five core areas: clinical skills, educational ability, management capacity, research capability, and professional development. The evaluation process consisted of both a self-assessment conducted by the specialist nurse and a formal review by the department nurse manager. The assessment questionnaire was developed by the hospital's Nursing Education Department using the Delphi method.

### 2.2 Practice of IBD specialist nurses under the SDM model

**Figure 1:**
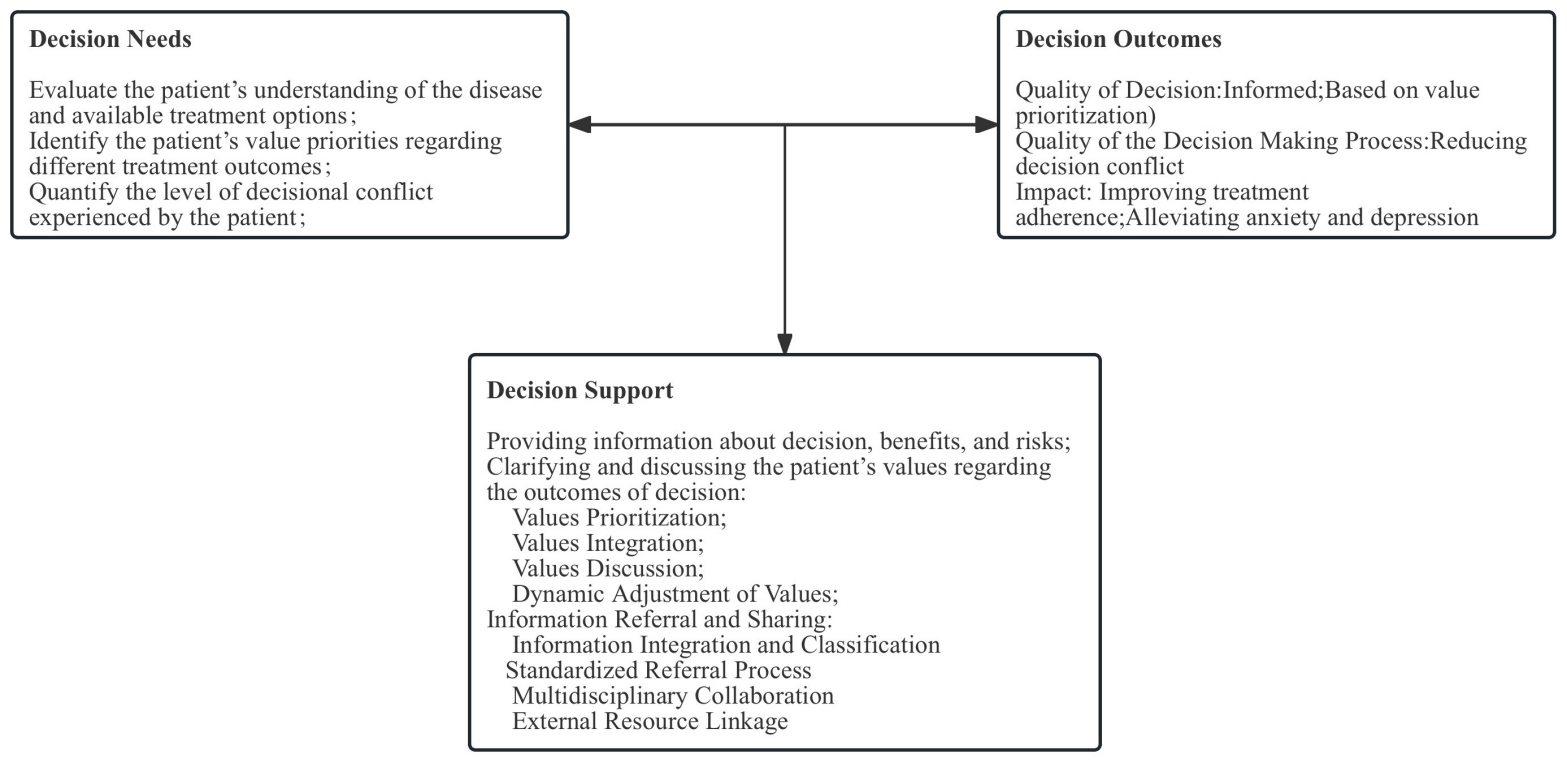
Practice model of IBD specialist nurses under the SDM model.

Building on this foundation, the IBD specialist nurse optimized and adapted the model according to clinical nursing practice to better address patient needs (Figure 1).

#### 2.2.1 Assessment of patient decision needs

Guided by the ODSF, the assessment of patient decision needs focused on reducing decisional conflict in patients with IBD through knowledge support, values clarification, and enhancement of decision readiness. The primary objectives of decision needs assessment by the IBD specialist nurse were to evaluate the patient's understanding of the disease and available treatment options; identify the patient's value priorities regarding different treatment outcomes (e.g., quality of life, safety, and cost); and quantify the level of decisional conflict experienced by the patient, such as hesitation, anxiety, or confusion due to contradictory information. The specific implementation was as follows.

##### 2.2.1.1 Initial screening

At the patient's first visit, the IBD specialist nurse utilized the Chinese version of the Decisional Conflict Scale (DCS) (12) to assess the patient's levels of uncertainty, confusion, and decisional conflict, where a lower score indicated less conflict. Demographic factors, such as age and education level, were also considered to predict potential variations in decision-making needs and to identify high-risk patients who demonstrate high decision conflict and limited disease knowledge. This study was approved by the Ethics Committee of the Second Affiliated Hospital, School of Medicine, Zhejiang University (Hangzhou, Zhejiang Province, China). The Institutional Review Board approved the informed consent process. Every participant signed the written informed consent form before the study began.

##### 2.2.1.2 In-depth assessment

(1) Knowledge assessment: based on the “treatment needs pyramid” for IBD patients, which includes needs such as endoscopic, histological, and clinical remission, the IBD specialist nurse assessed the patient's phase (e.g., active disease, remission, or perioperative period) and evaluated their understanding of IBD progression and perceptions of treatment side effects through a structured question-and-answer format.
(2) Value discussion: the nurse encouraged patients to prioritize short-term treatment goals, such as achieving rapid symptom relief, avoiding surgical intervention, and reducing healthcare costs, to help determine the urgency and importance of different treatment decisions. Based on the results of the initial screening and in-depth assessment, patients' decision needs were categorized into three dimensions, informational, value-based, and supportive, to provide precise and individualized decision support (Table 1).

**Table 1:** Classification and management of decision-making needs in IBD patients.

**Category**	**Goal**	**Specific support**
Informational needs	Provide information on disease treatment, follow-up, prognosis, etc.	Provide information focused on treatment and care alternatives, along with possible outcomes, based on the patient's personal values and preferences to facilitate decision-making.
Value-based needs	Help clarify preferences and define personal values	In the IBD shared decision-making outpatient clinic, discuss the alignment between the patient's values and treatment options, offering appropriate support based on the needs.
Supportive needs	Actively introduce external resources to enhance decision-making capability	(1) Hospital social workers; (2) IBD volunteers; (3) professional psychologists; and (4) community healthcare providers.

#### 2.2.2 SDM information support

During the SDM process, the IBD specialist nurse played a critical role in providing patients with comprehensive and accurate information regarding the benefits and risks of various treatment options, assisting them in systematically weighing the advantages and disadvantages. Since February 2022, our hospital has established a specialized IBD nursing outpatient clinic, where the IBD specialist nurse offers one-on-one consultations. In addition to verbal education during consultations, the nurse also distributes SDM-specific educational materials tailored to different patient needs, as outlined below.

##### 2.2.2.1 Newly diagnosed patients

For patients diagnosed within the past year, early establishment of disease understanding and management awareness is emphasized. The IBD specialist nurse provided an IBD navigation card, introducing the multidisciplinary IBD team, clinic hours, online consultation access via the internet hospital, and IBD community contact information. The nurse also provided a first-visit toolkit, containing educational materials such as basic disease knowledge, a dietary management manual, IBD patient narratives, and follow-up management forms.

##### 2.2.2.2 Perioperative patients

For patients scheduled for surgery within the upcoming month, the IBD specialist nurse offered a surgery toolkit, which included preoperative preparation guidelines, strategies for postoperative relapse prevention, dietary management instructions, video tutorials on stoma care, and psychological support resources. This toolkit aimed to familiarize patients with the surgical process, reduce preoperative anxiety, and provide comprehensive guidance for postoperative recovery.

##### 2.2.2.3 Pregnancy and conception-stage patients

For female patients from the time of obtaining a marriage certificate until 42 days postpartum, the nurse provided resources to assist in disease management during preconception, pregnancy, and postpartum periods, aiming to minimize adverse outcomes due to inadequate disease control. Materials provided included the IBD pregnancy manual, recommendations for pregnancy visit schedules, and a pocket guide titled 120 questions about ulcerative colitis and Crohn's disease: a handbook for inflammatory bowel disease patients (13).

##### 2.2.2.4 Biologic treatment patients

For patients initiating or receiving biologic therapies, the nurse provided the team-designed IBD biologic treatment follow-up plan card, test management forms, and detailed guidelines regarding biologic treatment processes, potential risks, and important precautions. These materials aimed to promote patient engagement in treatment adherence and enable early identification of adverse drug reactions.

##### 2.2.2.5 Patients requiring vaccination

Recognizing that vaccination can significantly reduce the incidence of opportunistic infections in patients with IBD, the IBD specialist nurse, referring to the ECCO Vaccination Guidelines (14) and the U.S. Vaccine Checklist (15), has designed a vaccination assessment form. The nurse assesses each patient's condition, treatment plan, and vaccination history to provide individualized vaccination recommendations based on immune suppression status.

#### 2.2.3 Clarification of patient values

Patient values are central to the decision-making process. The IBD specialist nurse plays a pivotal role in assisting patients to clarify their values and align them with appropriate treatment options. The specific implementation strategies were as follows.

##### 2.2.3.1 Values prioritization

The IBD specialist nurse prepared cards representing common values associated with IBD treatment, such as “Preserving bowel function,” “Minimizing treatment side effects,” “Ensuring treatment does not affect fertility,” “Reducing treatment costs,” and “Achieving rapid symptom relief.”

Patients were asked to rank these values in order of personal importance. The nurse then discussed the patient's rankings, exploring the reasoning and factors influencing their choices. This dialogue helped patients further clarify and organize their values. Following the discussion, patients selected their three most important core values, which were recorded for reference to aid subsequent treatment decisions.

##### 2.2.3.2 Values integration

Based on the patient's prioritized values (e.g., quality of life, treatment cost, and risk of side effects), and in collaboration with the IBD specialist's clinical evaluation, the IBD specialist nurse listed all viable treatment options, such as biologic therapy, surgery, and traditional immunosuppressive therapy. Each option was analyzed in terms of how well it aligns with the patient's core values. Utilizing a team-developed “values–treatment match table,” the nurse systematically matched treatment options with patient priorities. In cases with conflicts, the nurse carefully explained the reasons for the conflict and discussed potential alternative solutions to facilitate informed decision-making.

##### 2.2.3.3 Values discussion

The IBD specialist nurse provided detailed explanations regarding the potential side effects of each treatment option and discussed management strategies and follow-up care requirements. For instance, using a customized follow-up card, the nurse outlined the specific evaluations required at 2-, 6-month, and annual intervals. During consultations at the IBD SDM outpatient clinic, the nurse guided discussions among patients, their family members, friends, and healthcare team members, focusing on treatment goals, lifestyle adjustments, and family roles and responsibilities. Patients were given sufficient time to reflect on and, if necessary, adjust their value priorities. Throughout this process, the nurse actively communicated with the patient, promptly addressed any questions, and collected feedback.

##### 2.2.3.4 Dynamic adjustment of values

Recognizing that patient values may evolve due to changes in disease status, treatment response, or psychological wellbeing, the IBD specialist nurse reassessed patient values during follow-up visits every 2 months. Emotional and psychological states were evaluated using self-assessment scales for anxiety and depression. For patients presenting with mild-to-moderate anxiety and/or depression, access to the hospital's IBD team-developed online positive psychological intervention courses was provided to help alleviate stress. For patients with severe anxiety and/or depression, referral to a psychological specialist was recommended to ensure comprehensive care.

#### 2.2.4 Referral and information sharing

**Figure 2:**
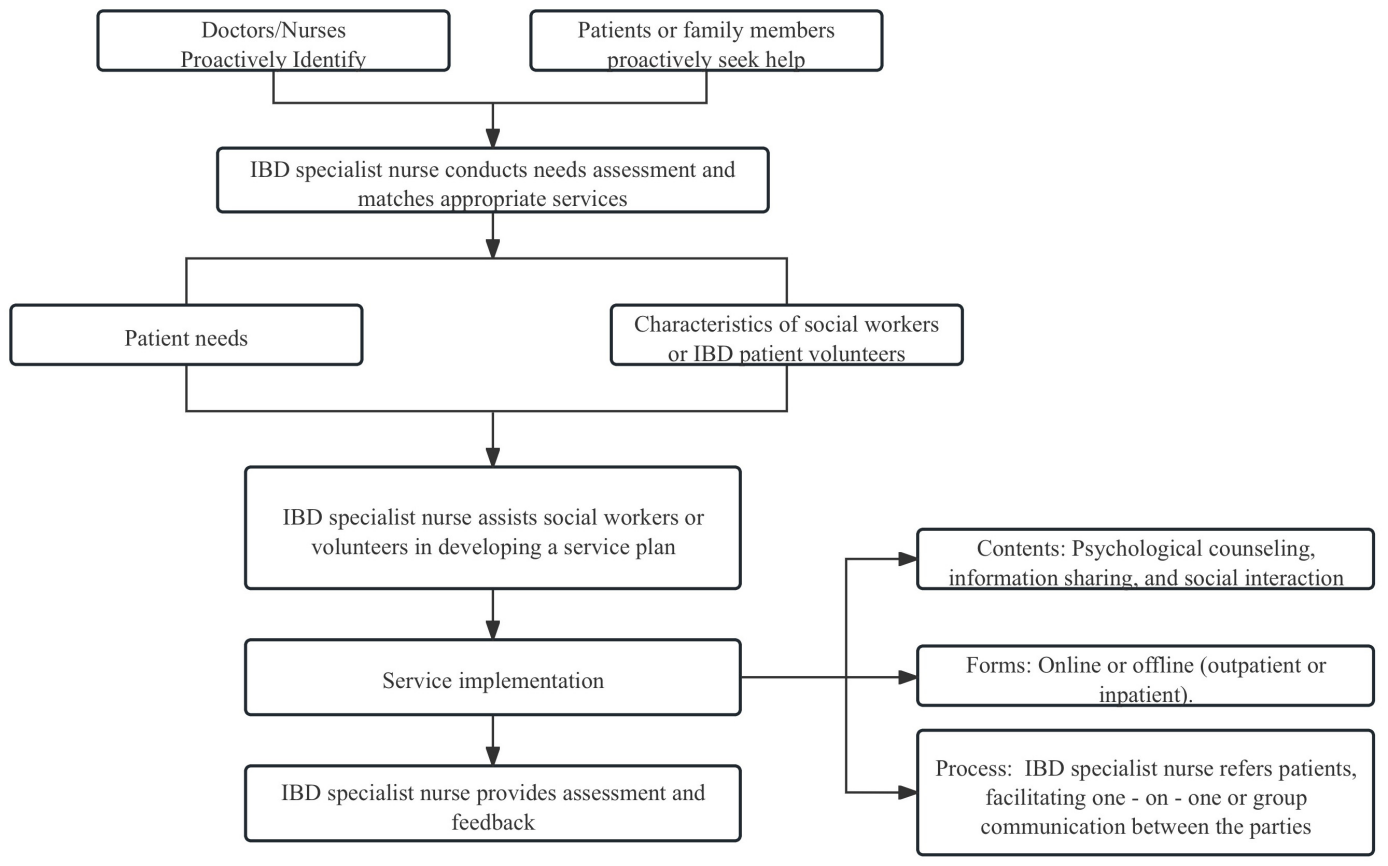
Process of social worker and IBD patient volunteer involvement.

Referral and information sharing are essential for ensuring that patient needs are met through coordinated multidisciplinary treatment plans. As the central information hub, the IBD specialist nurse facilitated the efficient integration and precise transmission of patient data through standardized processes, multidisciplinary collaboration, and the use of digital tools. The specific implementation strategies were as follows. The nurse utilized a social support assessment scale to evaluate the patient's current support system and determine the need for additional support from a social worker or IBD volunteer peers. The process for involving social workers and patient volunteers is presented in Figure 2.

##### 2.2.4.1 Information integration and classification

The IBD specialist nurse was responsible for integrating and classifying each patient's multidimensional data, which were recorded in the hospital's IBD registry database. These data included clinical information, decision-making needs, and socio-psychological assessments, collected at the initial consultation and at 2-, 6-, 12-month, and annual follow-ups. Clinical data fields are primarily based on the standard data set for inflammatory bowel disease (2021 edition) (16). Decision-making needs were assessed using the DCS and values clarification tools to identify the patient's priority goals and core needs in relation to treatment decisions. Socio-psychological data included measures of quality of life, anxiety and depression scores, and social support ratings.

##### 2.2.4.2 Standardized referral process

The IBD specialist nurse first evaluated the need for referral based on patient complaints or the collected data. If referral was indicated, the nurse issued a referral card to guide the patient to the appropriate department, such as the Pathology, Nutrition, or Psychological Services Department. The referral form documented the patient's decision conflict points and specific issues requiring specialist input. The nurse then ensured that the referral was completed on the same day, thereby closing the referral loop and guaranteeing that patients received timely and appropriate support.

##### 2.2.4.3 Multidisciplinary collaboration

The IBD specialist nurse coordinated the multidisciplinary team case discussions. Prior to each discussion, the nurse prepared and circulated a discussion agenda, defined the meeting objectives, and invited relevant specialists from Radiology, Pathology, Nutrition, and other departments. During the discussion, the nurse ensured that patient concerns were clearly articulated and that accurate information was conveyed to all participants. Afterward, the nurse summarized and recorded the meeting outcomes, serving as a basis for subsequent treatment planning.

##### 2.2.4.4 External resource linkage

In collaboration with the China inflammatory bowel disease foundation (17), the IBD specialist nurse facilitated patient access to specialized IBD communities, including groups for newly diagnosed patients, pregnant patients, stoma patients, university students, and families of adolescents. Participation in these peer support groups helped alleviate psychological stress. Furthermore, the nurse strengthened coordination with community healthcare resources by assisting patients in remission to continue maintenance therapy and follow-up care at nearby community hospitals, ensuring continuity of medical support across all stages of disease management.

## 3 Results

### 3.1 Service volume

Between March 2021 and March 2024, the IBD specialist nurse managed a total of 2,028 IBD patient visits, comprising 1,352 visits for patients with Crohn's disease and 676 visits for patients with ulcerative colitis. Among these visits, there were 1,521 newly diagnosed patients, 322 perioperative patients, 32 patients in the preconception or pregnancy stages, 1,632 patients receiving biologic therapy, and 544 patients requiring vaccination services.

Given that decision conflict is commonly observed during surgical decision-making for IBD patients, we assessed decision conflict levels in 322 perioperative patients at our institution (Table 2). The decision conflict levels observed were lower than those reported by Rini et al. (18).

**Table 2:** Decision conflict in surgical decision-making among study participants (*n* = 322).

**Variable**	**Group**	**Frequency**	**Percentage (%)**
Decision conflict	No decision conflict (< 25)	197	61.18
	Moderate decision conflict (25–37.5)	79	24.53
	Decision delay (>37.5)	46	14.29

### 3.2 Team members' recognition of IBD specialist nursing practice

A satisfaction questionnaire developed by our team was used to evaluate the recognition of the IBD specialist nurse's role across multiple dimensions, including role effectiveness, attitude and communication skills, professional competence, collaboration skills, research and information management abilities, decision-making capacity, and overall recognition. The questionnaire employed a 5-point Likert scale (ranging from 1 to 5, with higher scores indicating greater recognition) and was administered quarterly and annually. All hospital nurses participated in the evaluation. The results indicated that the overall annual recognition of the IBD specialist nurse's practice reached 94.12%.

### 3.3 Development of the IBD nursing discipline

Over the past 3 years, the IBD specialist nurse has actively contributed to the development of the hospital's IBD services by assisting in the establishment of five relevant protocols, revising two treatment guidelines, and improving 32 health education materials. The nurse also contributed to the design of eight standardized templates for disease course documentation and medical orders.

In terms of patient education and follow-up, the IBD specialist nurse provided health education to over 1,783 individuals annually within the hospital and to more than 150 individuals in the community. The nurse conducted follow-up care for over 2,683 discharged patients each year.

Additionally, the IBD specialist nurse provided training to more than 100 in-service nurses and nursing students annually, as well as over 80 gastroenterology residents and fellows. The nurse has actively participated in multiple quality improvement initiatives, including the “Improving Health Education Awareness for Newly Diagnosed IBD Patients,” “Shortening Pre-Hospital Preparation Time for IBD Patients on Biologic Therapy,” and “Improving the Implementation of Health Education for Perioperative IBD Patients” projects.

To date, the IBD specialist nurse has published four English-language papers as the first or co-author, obtained one utility model patent, and led or participated in three provincial-level research projects and one hospital-level project. Furthermore, the nurse contributed to the writing of two popular science books and received one provincial-level award.

## 4 Discussion

IBD is a complex, chronic condition characterized by a prolonged disease course, frequent relapses, and the need for multisystem and multidisciplinary management (19). In European countries, the critical role of the IBD specialist nurse and corresponding management protocols have been clearly defined and widely recognized (7). Within the SDM framework, the professional autonomy and active involvement of nurses are key components. However, research indicates that the participation of nurses in the decision-making process remains relatively limited, thereby constraining their potential contributions to SDM (20).

This study demonstrated, through a systematic practice process, the indispensable role of the IBD specialist nurse within the SDM model. In clinical practice, the IBD specialist nurse rapidly identifies high-risk patients and provides targeted support for treatment preparation through initial screening and in-depth assessments based on patients' specific needs. By delivering comprehensive, accurate, and easily understandable information regarding treatment options, the nurse empowers patients to make informed decisions while fully respecting their preferences, needs, and values. Moreover, the IBD specialist nurse plays a guiding role in clarifying patients' values and dynamically adjusting treatment plans to ensure alignment with personal goals and priorities.

Building on this foundation, the nurse facilitates multidisciplinary collaboration and information exchange, thereby forming a comprehensive patient management model. This model not only strengthens medical support but also integrates social and psychological resources. As a result, it enhances patient satisfaction, optimizes the allocation of medical resources, reduces the need for patients to seek consultations across multiple departments, and improves the overall efficiency of healthcare delivery.

A scientific role evaluation system is essential to ensuring the effective operation of the IBD specialist nurse position. Nevertheless, the current evaluation systems remain incomplete, lacking unified standards and quantifiable indicators, particularly concerning the professional contributions of nurses to the SDM process. Some studies have developed evaluation frameworks using literature reviews, semi-structured interviews, and the Delphi technique, covering domains such as theoretical knowledge, practical skills, and professional attitudes (21). However, these approaches are often limited in their ability to fully capture the nurse's contributions to patient decision support, values clarification, and multidisciplinary collaboration. This gap may contribute to inadequate nurse participation in decision-making, ultimately affecting both the quality of patient care and the effectiveness of clinical decisions.

Currently, evaluation tools specific to SDM competency among nurses are still under development, both domestically and internationally. A competency scale for oncology nurses engaged in SDM (SDM-N) has been established abroad, encompassing four dimensions: knowledge, attitude, communication skills, and adaptability (22). In domestic research, it has been reported that nurses' SDM abilities are generally at a moderate level, with influencing factors including years of clinical experience, participation in relevant training, empathy skills, and the degree of organizational support (23). However, there remains a lack of targeted investigation into the SDM competencies of IBD specialist nurses.

Thus, the construction of an evaluation system for IBD specialist nurses should adhere to a multidimensional approach to enhance both scientific rigor and practical applicability. Such an evaluation could encompass four main components. The first component is decision support effectiveness, focusing on the ability to accurately identify patients' decision-making needs, systematically provide evidence-based information, and guide value balancing. The second component is information management quality, emphasizing the establishment of standardized processes for information integration to ensure the completeness of patient data and the timeliness of interdisciplinary referrals. The third component is multidisciplinary collaboration effectiveness, evaluating communication and coordination skills within the team, as well as the efficiency of resource allocation. The fourth component is health outcomes improvement, involving quantitative assessment of indicators such as treatment adherence, symptom control rates, and quality of life scores, thereby forming a closed-loop evaluation mechanism. Through the implementation of such measures, a more comprehensive evaluation of the professional contributions of IBD specialist nurses can be achieved, providing a stronger scientific foundation for their career development.

Although the IBD specialist nurse plays a vital role within the SDM model, the training and promotion of IBD specialist nurse positions in China continues to face significant challenges. Currently, the training and certification system for specialist nurses organized by the Chinese Nursing Association does not include IBD specialist nurses. The absence of formal certification may restrict the scope of practice and diminish the professional authority of IBD specialist nurses in clinical settings, thereby affecting their effectiveness in multidisciplinary collaboration. Consequently, this limitation may hinder nurses' participation in SDM, partly due to the unclear definition of the nurse's role in clinical practice (24).

Internationally, the concept of SDM nurses, also known as decision coaches, originated in the mid-1970s within oncology specialties. As the traditional paternalistic doctor–patient relationship gradually evolved toward an SDM model, patients assumed a more active role in treatment decisions, promoting a patient-centered approach to care (25). In recent years, decision coaches have been instrumental in supporting patients with chronic conditions, such as diabetes, assisting them in understanding insulin administration methods and weighing potential risks and benefits through personalized decision-making support (26). Similarly, in breast cancer care, decision coaches have helped patients evaluate the trade-offs between breast-conserving surgery and mastectomy using decision-support tools, leading to improved decision-making quality and reduced preoperative anxiety (27).

Drawing on international experiences, training programs for IBD specialist nurses based on SDM principles could be developed in China, covering core competencies such as decision-support theory, communication skills, and patient preference assessment. In addition, policy guidance and financial incentives could encourage healthcare institutions to establish formal IBD specialist nurse positions, fostering close collaboration between IBD specialist nurses and multidisciplinary teams, including gastroenterology, surgery, nutrition, and psychology, to create a comprehensive patient management model.

Despite the demonstrated global clinical value of SDM in IBD care, its implementation within the Chinese healthcare system remains at an early stage. Barriers to effective SDM include information asymmetry: patients have insufficient understanding of their condition, treatment options, and potential risks, or clinicians fail to explain complex medical information in plain language;power imbalance: traditional paternalistic care models allow clinicians to dominate decisions, while patients, deferring to authority, hesitate to voice personal preferences; lack of standardized questionnaires, visual risk charts, or electronic decision aids, which reduces the efficiency of clinician-patient communication; and cultural factors: patients may avoid participation due to cultural norms such as reluctance to hear bad news or family-centered decision-making traditions. To operationalize this approach, healthcare institutions could integrate SDM into their nursing quality evaluation systems and use internal incentive mechanisms to encourage active nurse participation in the decision-making process. Nursing leadership should recognize the critical contributions of nurses in SDM, empowering them with greater professional autonomy and responsibility. Additionally, institutions could begin by introducing simple decision-support tools or small-scale pilot programs, gradually moving toward hospital-wide implementation. Regular evaluations of the SDM model's effectiveness should be conducted, with strategic adjustments made to better address the evolving needs of both patients and nursing teams. Furthermore, the use of digital tools, such as patient management platforms and intelligent decision-support systems, could facilitate the integration of patient information and enhance decision-making efficiency. Future initiatives should focus on training and education to establish a competency-based development model for IBD nurses' involvement in shared decision-making. This includes setting up SDM nursing centers or professional societies and developing continuing-education curricula for IBD nurses, with an emphasis on evidence-based practice, communication skills, and humanistic care. Ultimately, a core-competency framework anchored in nurses' roles and functions within SDM should be established.

In summary, by establishing the IBD specialist nurse position based on the SDM framework, our hospital explored the role and practical impact of specialist nurses in managing IBD patients. Through systematic assessments, information support, values clarification, and multidisciplinary collaboration led by IBD specialist nurses, patients experienced reduced decision conflict, improved treatment adherence, and enhanced quality of life. This approach offered innovative ideas and practical pathways for improving IBD patient management. Moving forward, by refining role evaluation systems, optimizing training and promotion mechanisms, and strengthening policy support, we aim to further enhance the professional capacity and reach of IBD specialist nurses, promote high-quality development in IBD disease management, and ultimately provide patients with better healthcare services.

## Data Availability

The original contributions presented in the study are included in the article/supplementary material, further inquiries can be directed to the corresponding author.
